# Supplementary material for: Can a nation-wide e-cohort of ADHD and ASD in childhood be established using Welsh routinely available datasets?
Source: BMJ Open. 2023 Aug 21;13(8):e071851. doi: 10.1136/bmjopen-2023-071851 (PMC10445352; doi:10.1136/bmjopen-2023-071851)
Supplement: Supplementary data [file bmjopen-2023-071851supp001.pdf]

Supplementary Tables:

Table s1: Details on the datasets utilised in this study:

Dataset	Description	Coverage	Timescale
The Welsh Demographics Service (WDS)	Register of all individuals who have ever had contact with the NHS or registered with a Welsh GP.	All individuals in Wales	Whole of the study period
Welsh Index of Multiple Deprivation (WIMD)	Dataset assigning a deprivation score derived from eight domains including employment, income and education to all Lower Super Output Areas (LSOAs; geographical areas comprising of around 1500 individuals). Individuals are assigned a deprivation index based on the LSOA of their current address in WDS.	All individuals in Wales	Whole of the study period
General Practice Database (GPD)	Attendance and clinical information for all primary care contacts. Includes diagnoses, symptoms and prescriptions.	79% of individuals in Wales from 333/432 GP practices	Whole of the study period
Patient Episode Database for Wales (PEDW)	Attendance and clinical information for all NHS Wales hospital admissions (both inpatient and day cases). Includes diagnoses, and specialty.	All individuals in Wales	Whole of the study period
Emergency Department Dataset	Administrative and clinical information for all NHS Wales Accident and Emergency Department attendances.	All individuals in Wales	Data available since 2009

Full details of these datasets are available at www.saildatabank.com

Langley K, et al. BMJ Open 2023; 13:e071851. doi: 10.1136/bmjopen-2023-071851

Table s2: List of read codes, ICD-10 codes and prescription codes utilised to identify ADHD and ASD:

Concept	Read codes and prescription codes	ICD-10 codes
ADHD	**Eu900, 9Ngp.., Eu9y7, E2E01, EU900, 6A61, 8BPT.., EU901, EU90.., E2E.., Zs91.00, zS91.11, ZS91.12, dc1.., dw1.., dw2.., dw3..,**	**F90, F90.0, F90.1, F90.2, F90.8, F90.9**
ASD	**E140, Eu840, Eu841, Eu845, Eu84z-1, 1J9**	**F84.0, F84.5, F84.9**

Langley K, *et al. BMJ Open* 2023; 13:e071851. doi: 10.1136/bmjopen-2023-071851

Table S3: Cox's regression analysis: **ADHD associations with outcomes**

	Model 1		Model 2		Model 3	
	**HR**	**95% CI**	**HR**	**95% CI**	**HR**	**95% CI**
**Anxiety/depression**						
**ADHD**	**2.36**	**2.20, 2.53**	**2.36**	**2.20, 2.53**	**2.32**	**2.17, 2.50**
Sex			0.44	0.41, 0.47	0.44	0.41, 0.47
Deprivation**					1.05	1.02, 1.07
**Self-harm**						
**ADHD**	**5.70**	**5.07, 6.40**	**5.70**	**5.06, 6.39**	**5.52**	**4.91, 6.20**
Sex			0.53	0.47, 0.60	0.43	0.47, 0.60
Deprivation**					1.12	1.08, 1.17
**Alcohol use**						
**ADHD**	**3.95**	**3.42, 4.56**	**3.95**	**3.42, 4.56**	**3.85**	**3.33, 4.44**
Sex			1.07	0.88, 1.28	1.06	0.88, 1.28
Deprivation**					1.10	1.05, 1.16
**Drug use**						
**ADHD**	**5.88**	**5.08, 6.80**	**5.88**	**5.09, 6.81**	**5.68**	**4.90, 6.57**
Sex			1.49	1.21, 1.83	1.49	1.21, 1.83
Deprivation**					1.15	1.09, 1.21
Emergency Department**room use**						
**ADHD**	**1.36**	**1.31, 1.41**	**1.36**	**1.31, 1.41**	**1.34**	**1.30, 1.39**
Sex			0.96	0.92, 1.00	0.96	0.92, 1.00
Deprivation**					1.04	1.02, 1.05
**Any primary care use**						
**ADHD**	2.63	2.46, 2.80	2.62	2.46, 2.80	2.58	2.42, 2.76
Sex			0.50	0.46, 0.53	0.50	0.46, 0.53
Deprivation**					1.06	1.03, 1.08
**Any hospital use (inc ED)**						
**ADHD**	1.36	1.31, 1.41	1.36	1.31, 1.41	1.35	1.30, 1.40
Sex			0.95	0.91, 0.99	0.95	0.91, 0.99
Deprivation**					1.03	1.02, 1.05

Models incrementally adjusting for covariates. * Age at end of follow up period as time variable; **WIMD quintile

Langley K, *et al. BMJ Open* 2023; 13:e071851. doi: 10.1136/bmjopen-2023-071851

Table s4: Binomial analysis of number of recorded incidents: ADHD and ASD associations with number of Anxiety/depression, self-harm and Emergency Department events

	**n**	**B**	**St. error**	**95% CI**
**ADHD**				
**Number of self-harm events**	1216	0.43	0.004	0.35, 0.51
**Number of Emergency Department visits**	14916	0.53	0.009	0.51, 0.55
**ASD**				
**Number of self-harm events**	484	0.53	0.06	0.42, 0.64
**Number of Emergency Department visits**	7514	0.26	0.01	0.23, 0.29

* Controlling for sex, age at end of follow up, proportion of follow up and deprivation

Table s5: Cox's regression analysis: **ADHD associations with self-harm using GP or hospital records only**

	Model 1		Model 2		Model 3	
	**HR**	**95% CI**	**HR**	**95% CI**	**HR**	**95% CI**
**GP records**						
**ADHD**	**6.61**	**5.65, 7.72**	**5.72**	**4.88, 6.70**	**5.52**	**4.71, 6.47**
Sex			0.43	0.37, 0.50	0.43	0.36, 0.50
Deprivation**					1.14	1.08, 1.20
**Hospital records**						
**ADHD**	**7.07**	**5.91, 8.48**	**5.94**	**4.95, 7.13**	**5.76**	**4.80, 6.92**
Sex			0.44	0.37, 0.52	0.44	0.37, 0.52
Deprivation**					1.12	1.05, 1.19

Models incrementally adjusting for covariates. * Age at end of follow up period as time variable; ** WIMD quintile

Langley K, *et al. BMJ Open* 2023; 13:e071851. doi: 10.1136/bmjopen-2023-071851

Table s6: Cox's regression analysis: **ADHD associations with outcomes MALES**

	**Model 1**		**Model 2**	
	**HR**	**95% CI**	**HR**	**95% CI**
**Anxiety/depression**				
**ADHD**	**2.36**	**2.16, 2.57**	**2.32**	**2.13, 2.54**
Deprivation**			1.06	1.03, 1.09
**Self-harm**				
**ADHD**	**5.75**	**5.00, 6.61**	**5.53**	**4.80, 6.36**
Deprivation**			1.16	1.10, 1.22
**Alcohol use**				
**ADHD**	**3.70**	**3.16, 4.33**	**3.58**	**3.06, 4.19**
Deprivation**			1.13	1.07, 1.20
**Drug use**				
**ADHD**	**5.67**	**4.85, 6.62**	**5.47**	**4.67, 6.39**
Deprivation**			1.14	1.08, 1.21
Emergency Department **use**				
**ADHD**	**1.32**	**1.27, 1.38**	**1.31**	**1.26, 1.36**
Deprivation**			1.03	1.02, 1.04
**Any primary care use**				
**ADHD**	**2.70**	**2.50, 2.92**	**2.65**	**2.45, 2.87**
Deprivation**			1.07	1.04, 1.10
**Any hospital use (inc ED)**				
**ADHD**	**1.32**	**1.27, 1.38**	**1.31**	**1.26, 1.37**
Deprivation**			1.03	1.02, 1.04

Models incrementally adjusting for covariates. * Age at end of follow up period as time variable; ** WIMD quintile

Table s7: Cox's regression analysis: **ADHD associations with outcomes FEMALES**

	Model 1		Model 2	
	**HR**	**95% CI**	**HR**	**95% CI**
**Anxiety/depression**				
**ADHD**	**2.35**	**2.09, 2.65**	**2.34**	**2.07, 2.64**
Deprivation**			1.03	0.99, 1.07
**Self-harm**				
**ADHD**	**5.57**	**4.51, 6.88**	**5.50**	**4.45, 6.80**
Deprivation**			1.05	0.98, 1.130
**Alcohol use**				
**ADHD**	**5.45**	**3.84, 7.75**	**5.49**	**3.86, 7.80**
Deprivation**				
**Drug use**				
**ADHD**	**7.69**	**5.05, 11.71**	**7.41**	**4.86, 11.30**
Deprivation**			1.17	1.01, 1.35
Emergency Department **use**				
**ADHD**	**1.51**	**1.39, 1.63**	**1.49**	**1.38, 1.61**
Deprivation**			1.05	1.03, 1.08
**Any primary care use**				
**ADHD**	**2.47**	**2.20, 2.77**	**2.44**	**2.18, 2.75**
Deprivation**			1.04	0.99, 1.08
**Any hospital use (inc ED)**				
**ADHD**	**1.52**	**1.41, 1.64**	**1.50**	**1.39, 1.62**
Deprivation**			1.05	1.03, 1.08

Models incrementally adjusting for covariates. * Age at end of follow up period as time variable; ** WIMD quintile

Table s8: Cox's regression – associations between ADHD and outcomes, stratified by Welsh index of multiple deprivation (WIMD):

	**Model 1***		**Model 2****	
	**HR**	**95% CI**	**HR**	**95% CI**
**Anxiety/depression**				
WIMD 1:	2.34	1.93, 2.84	2.38	1.97, 2.89
WIMD 2:	2.97	2.47, 3.57	2.92	2.43, 3.51
WIMD 3:	2.41	2.04, 2.86	2.35	1.99, 2.78
WIMD 4:	2.10	1.81, 2.45	2.12	1.82, 2.47
WIMD 5:	2.20	1.95, 2.94	2.21	1.96, 2.50
**Self-harm**				
WIMD 1:	5.17	3.61, 7.39	5.26	3.68, 7.53
WIMD 2:	10.51	7.43, 14.86	10.43	7.37, 14.75
WIMD 3:	6.58	4.97, 8.71	6.41	4.85, 8.49
WIMD 4:	4.72	3.72, 5.99	4.75	3.75, 6.03
WIMD 5:	4.46	3.66, 5.43	4.47	3.67, 5.44
**Alcohol use**				
WIMD 1:	5.74	3.58, 9.23	5.78	3.60, 9.28
WIMD 2:	4.16	2.80, 6.14	4.15	2.81, 6.13
WIMD 3:	3.19	2.37, 4.29	3.19	2.37, 4.29
WIMD 4:	4.38	3.21, 5.96	4.37	3.21, 5.96
WIMD 5:	3.48	2.70, 4.49	3.48	2.70, 4.48
**Drug use:**				
WIMD 1:	5.40	3.50, 8.33	5.36	3.47, 8.27
WIMD 2:	8.06	5.23, 12.42	8.12	5.27, 12.51
WIMD 3:	5.62	4.01, 7.88	5.65	4.03, 7.92
WIMD 4:	6.61	4.74, 9.20	6.57	4.72, 9.16
WIMD 5:	4.75	3.75, 6.01	4.74	3.75, 6.01
**ED Use:**				
WIMD 1:	1.36	1.23, 1.50	1.36	1.23, 1.50
WIMD 2:	1.35	1.23, 1.48	1.35	1.23, 1.48
WIMD 3:	1.37	1.26, 1.48	1.36	1.26, 1.48
WIMD 4:	1.27	1.18, 1.37	1.27	1.18, 1.37
WIMD 5:	1.38	1.29, 1.47	1.38	1.29, 1.47
**Any primary care use:**				
WIMD 1:	2.41	2.00, 2.89	2.44	2.03, 2.93
WIMD 2:	3.29	2.77, 3.91	3.24	2.73, 3.85
WIMD 3:	2.74	2.36, 3.19	2.31	2.31, 3.12
WIMD 4:	2.42	2.11, 2.79	2.44	2.12, 2.81
WIMD 5:	2.42	2.16, 2.71	2.42	2.16, 2.72

Langley K, *et al. BMJ Open* 2023; 13:e071851. doi: 10.1136/bmjopen-2023-071851

**Any hospital use (inc ED)**				
WIMD 1:	1.36	1.24, 1.50	1.36	1.24, 1.50
WIMD 2:	1.36	1.23, 1.49	1.36	1.23, 1.49
WIMD 3:	1.37	1.26, 1.49	1.37	1.26, 1.48
WIMD 4:	1.28	1.18, 1.38	1.28	1.19, 1.38
WIMD 5:	1.38	1.29, 1.46	1.38	1.29, 0.99

* Unadjusted association with ADHD; ** Association with ADHD, controlling for Sex.

Langley K, *et al. BMJ Open* 2023; 13:e071851. doi: 10.1136/bmjopen-2023-071851

Table s9: Cox's regression analysis: ADHD associations with outcomes, sample with complete data coverage only

	Model 1		Model 2		Model 3	
	**HR**	**95% CI**	**HR**	**95% CI**	**HR**	**95% CI**
**Anxiety/depression**						
**ADHD**	**2.23**	**2.07, 2.40**	**2.23**	**2.07, 2.40**	**2.20**	**2.04, 2.37**
Sex			0.43	0.39, 0.46	0.43	0.39, 0.46
Deprivation**					1.05	1.02, 1.07
**Self-harm**						
**ADHD**	**5.46**	**4.83, 6.18**	**5.46**	**4.83, 6.17**	**5.30**	**4.68, 5.99**
Sex			0.53	0.46, 0.60	0.53	0.46, 0.60
Deprivation**					1.12	1.07, 1.17
**Alcohol use**						
**ADHD**	**3.81**	**3.29, 4.43**	**3.82**	**3.29, 4.43**	**3.71**	**3.19, 4.31**
Sex			1.06	0.87, 1.30	1.06	0.87, 1.30
Deprivation**					1.11	1.05, 1.17
**Drug use**						
**ADHD**	**5.59**	**4.80, 6.51**	**5.59**	**4.80, 6.51**	**5.39**	**4.62, 6.28**
Sex			1.52	1.22, 1.90	1.52	1.22, 1.90
Deprivation**					1.14	1.08, 1.21
Emergency Department **use**						
**ADHD**	**1.36**	**1.31, 1.41**	**1.36**	**1.31, 1.41**	**1.35**	**1.30, 1.40**
Sex			0.94	0.91, 0.99	0.95	0.91, 1.04
Deprivation**					1.03	1.02, 1.04
**Any primary care use**						
**ADHD**	2.46	2.30, 2.63	2.46	2.30, 2.63	2.42	2.26, 2.59
Sex			0.48	0.45, 0.52	0.48	0.45, 0.52
Deprivation**					1.06	1.03, 1.08
**Any hospital use (inc ED)**						
**ADHD**	1.36	1.32, 1.42	1.36	1.32, 1.42	1.35	1.30, 1.40
Sex			0.94	0.90, 0.98	0.94	0.90, 0.98
Deprivation**					1.03	1.02, 1.04

Models incrementally adjusting for covariates. * Age at end of follow up period as time variable; **WIMD quintile

Langley K, et al. BMJ Open 2023; 13:e071851. doi: 10.1136/bmjopen-2023-071851

Table S10: Cox's regression analysis: **ASD associations with outcomes**

	**Model 1**		**Model 2**		**Model 3**	
	**HR**	**95% CI**	**HR**	**95% CI**	**HR**	**95% CI**
**Anxiety/depression**						
**ASD**	**2.11**	**1.91, 2.34**	**2.14**	**1.93, 2.36**	**2.14**	**1.93, 2.63**
Sex			0.47	0.42, 0.52	0.47	0.42, 0.52
Deprivation**					1.06	1.03, 1.10
**Self-harm**						
**ASD**	**2.93**	**2.45, 3.50**	**2.96**	**2.48, 3.54**	**2.96**	**2.48, 3.54**
Sex			0.49	0.41, 0.59	0.50	0.41, 0.60
Deprivation**					1.10	1.03, 1.17
**Alcohol use**						
**ASD**	**1.19**	**0.91, 1.55**	**1.19**	**0.91, 1.55**	**1.19**	**0.91, 1.55**
Sex			1.19	0.88, 1.61	1.21	0.89, 1.63
Deprivation**					1.10	1.01, 1.20
**Drug use**						
**ASD**	**2.21**	**1.66, 2.95**	**2.20**	**1.65, 2.93**	**2.20**	**1.65, 2.94**
Sex			2.37	1.51, 3.73	2.42	1.54, 3.81
Deprivation**					1.19	1.07, 1.31
Emergency Department **use**						
**ASD**	**0.95**	**0.90, 1.00**	**0.95**	**0.90, 1.00**	**0.95**	**0.90, 1.00**
Sex			0.99	0.93, 1.04	0.99	0.94, 1.05
Deprivation**					1.05	1.04, 1.07
**Any primary care use**						
**ASD**	1.99	1.81, 2.19	2.01	1.82, 2.21	2.01	1.82, 2.22
Sex			0.50	0.46, 0.56	0.51	0.46, 0.56
Deprivation**					1.06	1.02, 1.10
**Any hospital use (inc ED)**						
**ASD**	0.96	0.91, 1.01	0.96	0.91, 1.01	0.96	0.91, 1.01
Sex			0.98	0.93, 1.04	0.99	0.94, 1.04
Deprivation**					1.05	1.04, 1.07

Models incrementally adjusting for covariates. * Age at end of follow up period as time variable; **WIMD quintile

Table s11: Cox's regression analysis: **ASD associations with self-harm using GP or hospital records only**

	**Model 1**		**Model 2**		**Model 3**	
	**HR**	**95% CI**	**HR**	**95% CI**	**HR**	**95% CI**
**GP records**						
**ASD**	**3.60**	**2.86, 4.53**	**3.25**	**2.58, 4.10**	**3.26**	**2.58, 4.10**
Sex			0.40	0.31, 0.50	0.40	0.32, 0.50
Deprivation**					1.09	1.002, 1.18
**Hospital records**						
**ASD**	**3.75**	**2.85, 4.93**	**3.32**	**2.52, 4.37**	**3.32**	**2.52, 4.37**
Sex			0.40	0.30, 0.52	0.40	0.30, 0.52
Deprivation**					1.03	0.93, 1.13

Models incrementally adjusting for covariates. * Age at end of follow up period as time variable; ** WIMD quintile

Langley K, et al. BMJ Open 2023; 13:e071851. doi: 10.1136/bmjopen-2023-071851

Table s12: Cox's regression analysis: **ASD associations with outcomes MALES**

	**Model 1**		**Model 2**	
	**HR**	**95% CI**	**HR**	**95% CI**
**Anxiety/depression**				
**ASD**	**2.39**	**2.10, 2.71**	**2.39**	**2.11, 2.72**
Deprivation**			1.08	1.03, 1.13
**Self-harm**				
**ASD**	**2.65**	**2.12, 3.32**	**2.66**	**2.12, 3.33**
Deprivation**			1.14	1.06, 1.24
**Alcohol use**				
**ASD**	**1.08**	**0.80, 1.46**	**1.08**	**0.80, 1.47**
Deprivation**			1.13	1.03, 1.24
**Drug use**				
**ASD**	**1.98**	**1.46, 2.70**	**1.99**	**1.46, 2.70**
Deprivation**			1.17	1.05, 1.31
Emergency Department **use**				
**ASD**	**0.90**	**0.84, 0.95**	**0.90**	**0.85, 0.95**
Deprivation**			1.06	1.04, 1.08
**Any primary care use**				
**ASD**	**2.21**	**1.95, 2.49**	**2.21**	**1.96, 2.49**
Deprivation**			1.08	1.03, 1.12
**Any hospital use**				
**ASD**	**0.91**	**0.90, 0.96**	**0.91**	**0.86, 0.97**
Deprivation**			1.06	1.04, 1.08

Models incrementally adjusting for covariates. * Age at end of follow up period as time variable; ** WIMD quintile

Langley K, *et al. BMJ Open* 2023; 13:e071851. doi: 10.1136/bmjopen-2023-071851

Table s13: Cox's regression analysis: **ASD associations with outcomes FEMALES**

	**Model 1**		**Model 2**	
	**HR**	**95% CI**	**HR**	**95% CI**
**Anxiety/depression**				
**ASD**	**1.78**	**1.50, 2.10**	**1.78**	**1.51, 2.10**
Deprivation**			1.04	0.98, 1.10
**Self-harm**				
**ASD**	**3.60**	**2.68, 4.83**	**3.60**	**2.68, 4.83**
Deprivation**			1.03	0.93, 1.15
**Alcohol use**				
**ASD**	**1.72**	**0.98, 3.02**	**1.72**	**0.98, 3.02**
Deprivation**			1.01	0.84, 1.23
**Drug use**				
**ASD**	**4.99**	**2.07, 12.05**	**5.05**	**2.09, 12.19**
Deprivation**			1.30	0.93, 1.81
Emergency Department **use**				
**ASD**	**1.16**	**1.04, 1.29**	**1.16**	**1.04, 1.29**
Deprivation**			1.04	1.01, 1.08
**Any primary care use**				
**ASD**	**1.70**	**1.44, 2.01**	**1.70**	**1.44, 2.01**
Deprivation**			1.03	0.98, 1.09
**Any hospital use (inc ED)**				
**ASD**	**1.15**	**1.04, 1.29**	**1.16**	**1.04, 1.29**
Deprivation**			1.05	1.01, 1.08

Models incrementally adjusting for covariates. * Age at end of follow up period as time variable; **Proportion of follow up period with valid records ***WIMD quintile

Table s14: Cox's regression – associations between ASD and outcomes, stratified by Welsh index of multiple deprivation (WIMD):

	**Model 1***		**Model 2****	
	**HR**	**95% CI**	**HR**	**95% CI**
**Anxiety/depression**				
WIMD 1:	2.13	1.66, 2.72	2.17	1.70, 2.78
WIMD 2:	2.14	1.64, 2.79	2.22	1.70, 2.89
WIMD 3:	2.56	2.04, 3.22	2.45	1.95, 3.07
WIMD 4:	1.96	1.58, 2.42	2.02	1.63, 2.50
WIMD 5:	1.98	1.63, 2.40	2.00	1.65, 2.42
**Self-harm**				
WIMD 1:	3.09	1.88, 5.09	3.17	1.93, 5.22
WIMD 2:	2.80	1.80, 4.37	2.91	1.86, 4.53
WIMD 3:	4.02	2.68, 6.04	3.81	2.54, 5.73
WIMD 4:	2.14	1.50, 3.06	2.20	1.53, 3.15
WIMD 5:	3.10	2.19, 4.38	3.12	2.20, 4.41
**Alcohol use**				
WIMD 1:	1.45	0.73, 2.89	1.45	0.73, 2.88
WIMD 2:	1.04	0.53, 2.00	1.03	0.54, 1.99
WIMD 3:	1.48	0.80, 2.74	1.46	0.79, 2.70
WIMD 4:	0.74	0.40, 1.37	0.73	0.39, 1.35
WIMD 5:	1.45	0.89, 2.34	1.44	0.89, 2.32
**Drug use:**				
WIMD 1:	1.53	0.65, 3.57	1.51	0.64, 3.53
WIMD 2:	2.12	1.01, 4.44	2.07	0.99, 4.34
WIMD 3:	3.34	1.52, 7.33	3.42	1.56, 7.52
WIMD 4:	2.45	1.45, 4.14	2.38	1.41, 4.02
WIMD 5:	1.88	1.09, 3.24	1.86	1.08, 3.22
**ED Use:**				
WIMD 1:	0.94	0.82, 1.07	0.94	0.82, 1.07
WIMD 2:	0.96	0.83, 1.09	0.96	0.84, 1.09
WIMD 3:	1.03	0.92, 1.15	1.02	0.91, 1.15
WIMD 4:	0.90	0.80, 0.99	0.89	0.80, 1.00
WIMD 5:	0.96	0.86, 1.06	0.95	0.86, 1.06
**Any primary care use:**				
WIMD 1:	1.96	1.54, 2.50	2.00	1.57, 2.54
WIMD 2:	1.88	1.47, 2.42	1.95	1.52, 2.51
WIMD 3:	2.38	1.91, 2.96	2.28	1.83, 2.85

WIMD 4:	1.94	1.58, 2.39	1.99	1.62, 2.45
WIMD 5:	1.89	1.57, 2.29	1.91	1.58, 2.30
**Any hospital use (inc ED)**				
WIMD 1:	0.94	0.83, 1.07	0.94	0.83, 1.08
WIMD 2:	0.97	0.85, 1.10	0.96	0.85, 1.10
WIMD 3:	1.04	0.93, 1.17	1.03	0.92, 1.16
WIMD 4:	0.91	0.82, 1.01	0.91	0.81, 1.01
WIMD 5:	0.96	0.86, 1.07	0.95	0.86, 1.07

* Unadjusted association with ADHD; ** Association with ADHD, controlling for Sex and proportion of follow up.

Langley K, *et al. BMJ Open* 2023; 13:e071851. doi: 10.1136/bmjopen-2023-071851

Table s15: Cox's regression analysis: **ASD associations with outcomes**

	**Model 1**		**Model 2**		**Model 3**	
	**HR**	**95% CI**	**HR**	**95% CI**	**HR**	**95% CI**
**Anxiety/depression**						
**ASD**	**2.00**	**1.81, 2.22**	**2.02**	**1.82, 2.24**	**2.02**	**1.82, 2.24**
Sex			0.46	0.41, 0.51	0.46	0.41, 0.51
Deprivation**					1.06	1.02, 1.09
**Self-harm**						
**ASD**	**2.96**	**2.45, 3.57**	**2.98**	**2.47, 3.60**	**2.98**	**2.47, 3.60**
Sex			0.46	0.38, 0.56	0.47	0.38, 0.57
Deprivation**					1.08	1.01, 1.15
**Alcohol use**						
**ASD**	**1.27**	**0.96, 1.68**	**1.27**	**0.96, 1.68**	**1.27**	**0.96, 1.68**
Sex			1.12	0.81, 1.54	1.13	0.82, 1.56
Deprivation**					1.10	1.00, 1.21
**Drug use**						
**ASD**	**1.95**	**1.43, 2.67**	**1.94**	**1.42, 2.65**	**1.94**	**1.43, 2.65**
Sex			2.23	1.38, 3.60	2.27	1.41, 3.67
Deprivation**					1.19	1.07, 1.33
Emergency Department **use**						
**ASD**	**0.95**	**0.90, 1.00**	**0.95**	**0.90, 1.00**	**0.95**	**0.90, 1.00**
Sex			0.97	0.92, 1.03	0.98	0.92, 1.03
Deprivation**					1.05	1.03, 1.06

**Any primary care use**						
**ASD**	**1.86**	**1.68, 2.06**	**1.87**	**1.69, 2.07**	**1.87**	**1.70, 2.07**
Sex			0.49	0.44, 0.54	0.49	0.44, 0.54
Deprivation**					1.05	1.02, 1.09
**Any hospital use (inc ED)**						
**ASD**	**0.96**	**0.91, 1.01**	**0.96**	**0.91, 1.01**	**0.96**	**0.91, 1.01**
Sex			0.97	0.92, 1.03	0.98	0.92, 1.03
Deprivation**					1.05	1.03, 1.06

Models incrementally adjusting for covariates. * Age at end of follow up period as time variable; **WIMD quintile

Supplementary table s16: Directly assessed ADHD hybrid cohort and e-cohort only sample comparisons:

	**Nested ADHD sample**	**e-cohort sample**	**OR (95% CI) or t**
**N**	154	9379	
	Mean (sd) or N (%)		
**Male gender**	134 (87.0)	7583 (80.90)	1.587 (0.99, 2.55)
**Age at end of follow up (GP records)**	19.11 (2.20)	21.20 (3.80)	t=11.320, p<0.001
**Deprivation index quintile**	3.435 (1.56)	3.421 (1.40)	t=-0.11, p=0.91
**Age at ADHD diagnosis: Mean (sd)**	8.41 (3.34)	10.57 (3.86)	t= 7.95 p<0.001
**Age at ASD diagnosis: Mean (sd)**	9.33 (5.44)	10.96 (5.09)	t=1.45, p=0.15
**With comorbid ASD: N (%)**	26 (16.90)	1340 (14.30)	t=1.36, p=0.19
**Anxiety/depression- any: N (%)**	15 (9.70)	1977 (21.10)	0.40 (0.24, 0.69)
**Anxiety/depression events: Mean (sd)**	0.14 (0.50)	0.50 (1.45)	t=8.223, p=<0.001
**Drug use - any: N (%)**	10 (6.50)	800 (8.50)	0.745 (0.39, 1.42)
**Drug use - events: Mean (sd)**	0.08 (0.34)	0.27 (2.50)	t=0.15, p=0.36
**Alcohol use – any: N (%)**	5 (3.20)	675 (7.20)	0.43 (0.18, 1.06)
**Alcohol use – events: Mean (sd)**	0.06 (0.37)	0.19 (1.71)	t=0.93, p=0.35
**Self-harm – any: N (%)**	6 (3.9)	1158 (12.3)	t=6.947, p<0.001
**Self-harm – events: Mean (sd)**	0.09 (0.50)	0.40 (1.91)	0.288 (0.13, 0.65)
**Emergency Department – any: N (%)**	98 (63.6)	6021 (64.2)	0.976 (0.701, 1.36)
**Emergency Department – events: Mean (sd)**	2.36 (3.81)	3.53 (7.18)	3.69, p<0.001

Langley K, *et al. BMJ Open* 2023; 13:e071851. doi: 10.1136/bmjopen-2023-071851

Supplementary figures:

Figure S1:

Databases and process to identify directly assessed ADHD cohort:

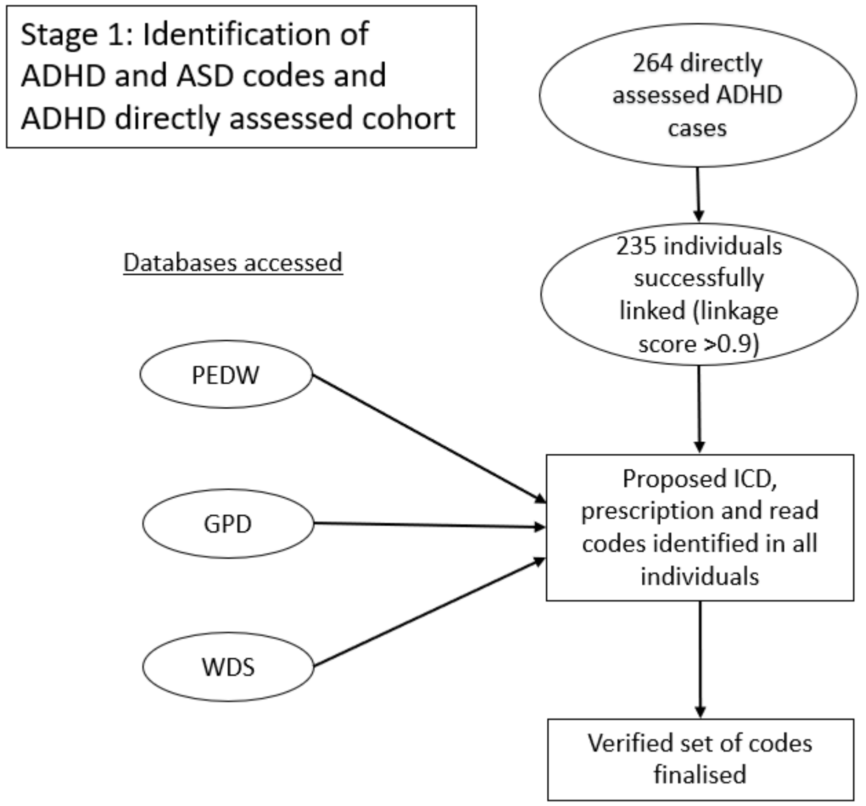

Abbreviations: ADHD= Attention Deficit Hyperactivity Disorder; GPD=General Practice Database; ICD= International Classification of Diseases; PEDW= Patient Episode Database for Wales; WDS=Welsh demographics Service.

Figure S2: Databases and process to identify individuals with ADHD and ASD:

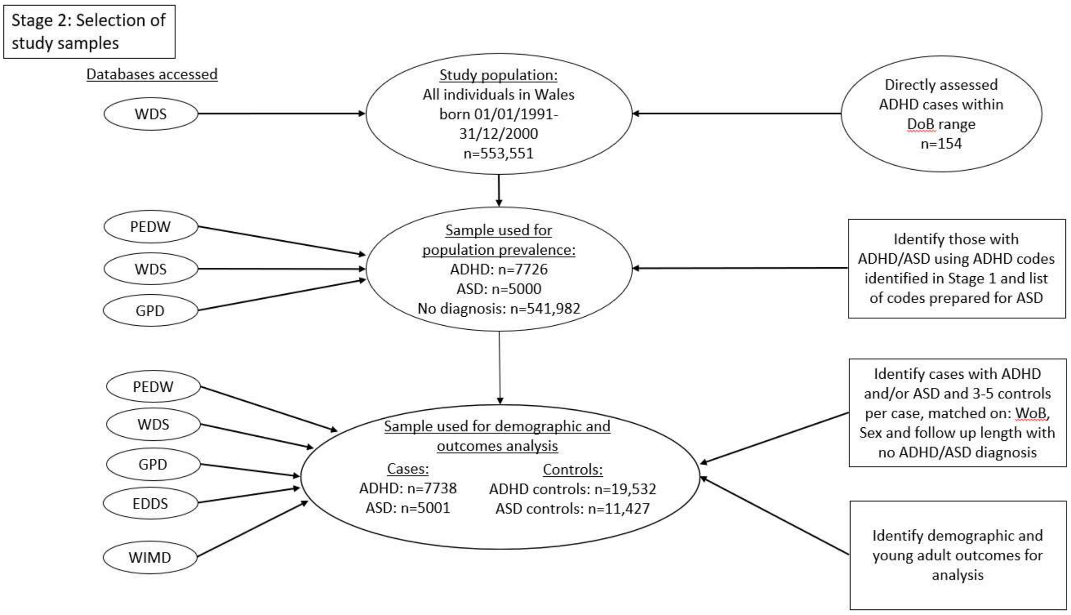

Abbreviations: ADHD= Attention Deficit Hyperactivity Disorder; ASD= Autism Spectrum Disorder; DoB=Date of birth; EDDS= Emergency Department Dataset; GPD=General Practice Database; PEDW= Patient Episode Database for Wales; WDS=Welsh demographics Service; WIMD=Welsh Index of Multiple Deprivation; WoB=Week of birth.

Figure S3:

Associations between ADHD and early adult outcomes, by deprivation index:

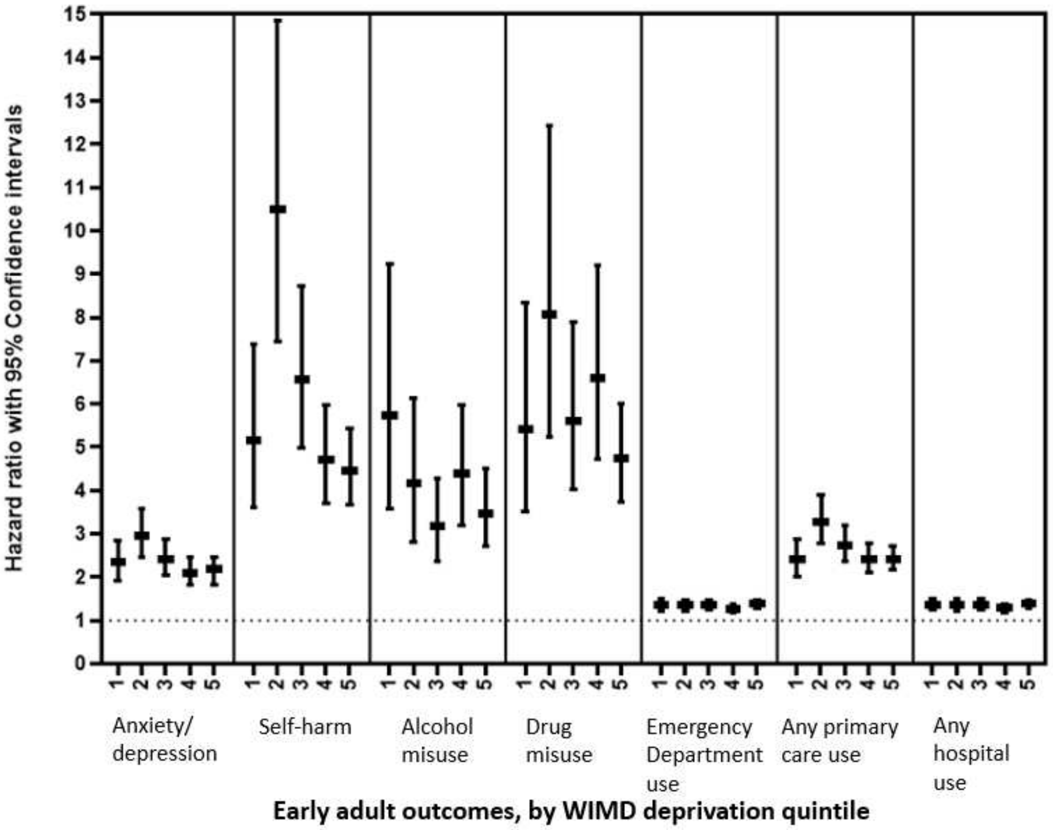

WIMD= Welsh index of multiple deprivation. Quintiles 1-5, level 5 represents the most deprived areas.

Langley K, *et al. BMJ Open* 2023; 13:e071851. doi: 10.1136/bmjopen-2023-071851

Supplementary Figure S4:

Associations between ASD and early adult outcomes:

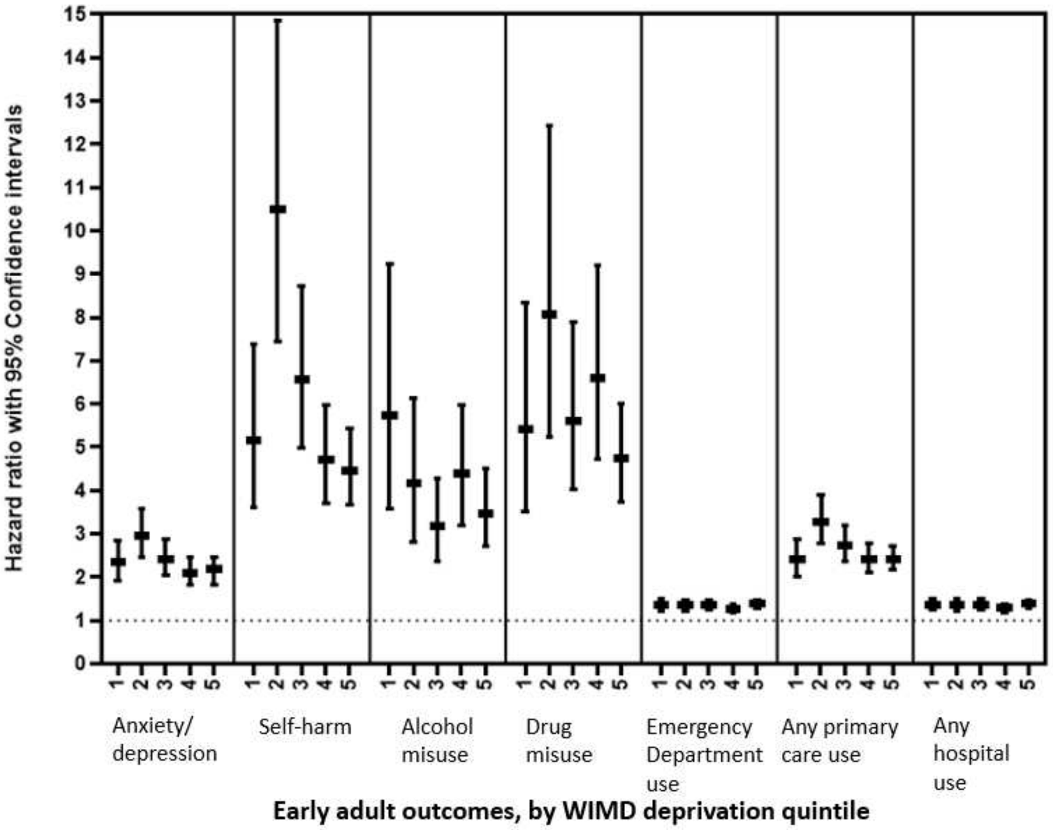

WIMD= Welsh index of multiple deprivation. Quintiles 1-5, level 5 represents the most deprived areas.

Langley K, *et al. BMJ Open* 2023; 13:e071851. doi: 10.1136/bmjopen-2023-071851
