# Supplementary material for: Can a nation-wide e-cohort of ADHD and ASD in childhood be established using Welsh routinely available datasets?
Source: BMJ Open. 2023 Aug 21;13(8):e071851. doi: 10.1136/bmjopen-2023-071851 (PMC10445352; doi:10.1136/bmjopen-2023-071851)
Supplement: Author's manuscript [file bmjopen-2023-071851.draft_revisions.pdf]

BMJ Open

BMJ Open is committed to open peer review. As part of this commitment we make the peer review history of every article we publish publicly available.

When an article is published we post the peer reviewers' comments and the authors' responses online. We also post the versions of the paper that were used during peer review. These are the versions that the peer review comments apply to.

The versions of the paper that follow are the versions that were submitted during the peer review process. They are not the versions of record or the final published versions. They should not be cited or distributed as the published version of this manuscript.

BMJ Open is an open access journal and the full, final, typeset and author-corrected version of record of the manuscript is available on our site with no access controls, subscription charges or pay-per-view fees (http://bmjopen.bmj.com).

If you have any questions on BMJ Open's open peer review process please email info.bmjopen@bmj.com
